# Strategies to enhance engagement in CBT for adult ADHD: an innovation tournament with stakeholders

**DOI:** 10.3389/fpsyt.2026.1824100

**Published:** 2026-09-18

**Authors:** Margaret H. Sibley, Melisa Shafiee, Laura E. Knouse

**Affiliations:** 1 Department of Psychiatry and Behavioral Sciences, University of Washington School of Medicine, Seattle, WA, United States; 2 Department of Psychology, University of Washington, Seattle, WA, United States; 3 Department of Psychology, University of Richmond, Richmond, VA, United States

**Keywords:** ADHD, cognitive behavioral therapy, intervention design, stakeholder co-design, treatment engagement

## Abstract

**Objective:**

Adults with ADHD report challenges engaging in cognitive-behavioral therapy (CBT). This study investigates practices that might be applied to improve intervention fit and therapeutic engagement of CBT for adults with ADHD.

**Method:**

We conducted an Innovation Tournament (IT) and member checking with participants (*N=*102) who were adults with ADHD and mental health practitioners who regularly treat adults with ADHD. Go-Zone plots were utilized to identify strategies with the highest feasibility and importance as well as strategies with low feasibility and/or importance that might be candidates for de-implementation. Barriers to CBT engagement and practitioner implementation of practices were also evaluated qualitatively.

**Results:**

A range of engagement barriers were identified, chief among them practitioner low understanding of ADHD (44.4%) and lack of attention to relevant impairments (33.3%). The highest rated focal areas for CBT were task management, organization, planning, and prioritization, emotion regulation strategies, responding to negative/maladaptive thoughts, anti-procrastination and self-motivation strategies, and long-term goal setting. Top recommendations for engagement strategies include ensuring high therapist knowledge of ADHD and 17 elements consistent with Motivational Interviewing. Some practices commonly found in CBTs for ADHD were rated as unpopular with stakeholders, most notably providing weekly ratings of ADHD symptoms as progress monitoring. Some practices rated as important to adults with ADHD were rated as not feasible to practitioners—most notably reducing shame and nurturing confidence. Implementation barriers identified were primarily related to low practitioner knowledge and skills, but also sometimes systemic constraints on practice.

**Conclusions:**

Implementation of recommended practices noted in this study should be tested empirically to understand the extent to which they increase engagement and effectiveness over standard practice.

## 1 Introduction

Cognitive-behavioral therapy (CBT) is one of two well-established treatment classes for attention deficit/hyperactivity disorder (ADHD) in adults, with dozens of randomized controlled trials (RCTs), systematic reviews, and meta-analyses supporting its efficacy for reducing core ADHD symptoms, managing emotional symptoms, and ameliorating functional impairments (1–8). CBT for adults with ADHD offers incremental efficacy over medication alone for improving core ADHD symptoms (9) with effects that maintain up to a year (10). Research-backed CBT protocols for adult ADHD are universally short-term (*M=*12 sessions), didactic, and standardized. Although they tend to include diverse content elements (11), they share common elements that include ADHD psychoeducation, executive function (EF) skills training, and cognitive restructuring that addresses maladaptive thoughts about ADHD and the self (11). Evidence syntheses find that EF skills and cognitions related to self-concept are the most robust mechanisms of change in ADHD symptom severity during adult ADHD CBT (10, 12).

Despite research trial efficacy, many adults with ADHD do not access CBT for ADHD in real world contexts. In the U.S., less than half of adults with ADHD are receiving any form of psychotherapy for their difficulties (13). This is not surprising, as availability of CBT for ADHD in the community is up to three times lower than for anxiety and depression (14) with half of psychologists who treat adults with ADHD reporting that they prioritize care for patients with other diagnoses (15). CBT also may have a negative reputation among adults with ADHD, with clients reporting that they view routine care CBT therapists as insufficiently skilled to adapt treatment to their struggles, unempathetic about their symptoms, and unable to tailor care to the client’s unique needs and personal goals (16). However, it should be noted that clients with ADHD often struggle with scheduling and session attendance, homework completion, follow-through on intentions, staying on topic in session, and sustain motivation to actively participate (17, 18). Thus, it is not surprising that some clinicians report that they find delivering CBT to adults with ADHD to be aversive (15).

Clinicians who provide CBT to adults with ADHD may currently lack population-specific strategies to support client engagement. Nearly half of psychologists report that they are not confident or only somewhat confident when treating adult ADHD, only a third received didactics in adult ADHD during their training, and only about half had treated an adult with ADHD by the conclusion of their post-doctoral training (15). In addition to low clinical exposure to adult ADHD broadly, there is yet to be an ADHD-specific toolbox of engagement-focused practices for clinicians who wish to improve their ability to work with adults with ADHD. Engagement-focused approaches have been applied widely in CBTs for adolescents with ADHD (19–22). Primarily, motivational interviewing (MI) (23) has received ADHD-specific adaptations and can be blended into CBTs for ADHD to increase interest and active participation in treatment, as well as follow-through on CBT homework assignments and intentions set during treatment (24). Therefore, it is possible that these ADHD-specific applications of MI might be applied to CBT delivered to adults with ADHD with good effect.

In addition to borrowing from the adolescent ADHD literature, collecting and validating adult ADHD-specific CBT engagement strategies could be enhanced by co-design with adults with ADHD and the practitioners who serve them. To achieve this aim, we conducted an Innovation Tournament (IT) (25) with adults with ADHD and practice experts to generate original ideas for making adult CBT models more ADHD-friendly, revisit the utility of existing adult ADHD CBT content, and explore developmental adaptations of ADHD-specific engagement strategies used with adolescents. ITs are a structured research method in which a group of participants generates, evaluates, and refines ideas in response to a common challenge (25). In the social sciences, they may commonly include a co-design process in which stakeholders generate ideas to solve a clinical problem in round 1, which are voted on by a broader set of stakeholders in round 2, and member checked in round 3. The findings of this study will be used to suggest a set of best practices to enhance engagement in CBT for adults with ADHD in routine care mental health contexts. These recommendations will include both new practices to implement and existing practices to potentially de-implement. Future work might test the effectiveness of these practices in enhancing CBT for adults with ADHD.

## 2 Methods

This study protocol was approved by the University of Washington Institutional Review Board (STUDY00024190) and all participants provided informed consent prior to participation.

### 2.1 Participants

#### 2.1.1 Innovation tournament rounds 1 and 2 sample

Participants were 69 adults with ADHD as well as 16 practice experts on adult ADHD CBT. Adults with ADHD were recruited from a registry of individuals who signed up to participate in adult ADHD research conducted at the University of Washington. To be eligible for the study, adults with ADHD were requited to be over the age of 18, reside in the United States, read English, and report being diagnosed with ADHD by a licensed healthcare provider. Adult ADHD practice experts were recruited from a professional listserv for individuals with a special interest in the diagnosis and treatment of Attention Deficit/Hyperactivity Disorder. To be eligible, practice experts were required to endorse that they were a licensed mental health care professional who routinely delivers psychotherapy to adults with ADHD and/or an academic expert on adult ADHD psychosocial treatment.

From the 69 participants, sixteen adults with ADHD participated in the IT’s initial idea generation step and fifty-three adults with ADHD provided importance ratings on the list of generated ideas. There was no participant overlap between rounds. The sixteen practice experts provided feasibility and importance ratings on the list of generated ideas. Characteristics of the adult ADHD samples are provided in **Table 1**. 81.3% of practice experts reported that they were practicing psychologists. Additionally, 31.3% reported that they were academic experts. One practice expert elected not to answer this question. Mean age of practice experts was 41.29 (*SD=*9.02) and they were 56.3% women, 75.0% White, non-Hispanic, 6.3% Latinx/Hispanic, 6.3% Multiracial, 6.3% Asian or Pacific Islander, and 6.3% preferred not to say.

**Table 1:** Characteristics of participating adults with ADHD.

Characteristic	Idea generation sample (*N*=16)	Validation sample (N=53)
Age *M*(*SD*)	44.53 (11.42)	47.16(13.22)
Gender (%)		
Man	12.5	18.9
Woman	81.3	77.4
Non-binary	6.3	3.8
Race/Ethnicity (%)		
Asian or Pacific Islander	12.5	3.8
Hispanic Any Race	0.0	7.5
Middle Eastern/North African	0.0	1.9
White Non-Hispanic	87.5	81.1
Prefer not to Disclose	0.0	5.7
Highest Education Level (%)		
Middle School or Equivalent	0.0	1.9
High School or Equivalent	12.5	13.2
Associates Degree	25.0	5.7
Bachelors Degree	31.3	35.8
Masters Degree or Higher	31.3	43.4
Age at ADHD Diagnosis *M*(*SD*)	32.81(14.82)	36.87(16.28)
Received ADHD Psychotherapy (%)	56.3	50.8
Satisfaction Level^a^ *M*(*SD*)	3.22(.97)	3.22(1.07)

^a^ Satisfaction Level with previous ADHD Psychotherapy was measured on a 1–5 scale from 1=very unsatisfied to 5=Very Satisfied.

#### 2.1.2 Member checking sample

The member checking sample included 17 adults who were required to either have been diagnosed with ADHD by a licensed healthcare provider (n=3) or be a licensed healthcare provider who routinely works with adults with ADHD (n=3) or both (n=11). Participants were recruited during a public presentation of the results of the IT’s idea generation and idea rating rounds and represented the attendees who were willing to complete a member checking survey after the public presentation. The member checking sample was 94.1% women, 88.2% White, and an average age of 48.56 (SD = 11.33). Practitioners were 7 master’s level therapists, 4 psychologists, 1 doctor of social work, and three physicians.

### 2.2 Procedures

All data in this study were collected via Qualtrics. Adults who had signed up for the research registry on adult ADHD were offered the opportunity to participate in the idea generation round of this study via email. In round I of the research 16 adults who provided electronic consent to participate were given a qualitative survey to generate ideas. Participants were asked to provide as many ideas as they could think of to answer these questions and were encouraged to consider “how to help clients want to keep coming back to sessions and to follow-through on their intentions for change and growth.”

All ideas generated by participants were subsequently listed on a rating scale. Redundant ideas were combined into single items. The investigators contributed 41 additional items to the scale: 20 common content elements of adult ADHD CBT that were not included on the list of adult suggestions (11), five CBT process elements extracted from traditional CBT manuals (6), and 16 ADHD-specific applications of MI processes, previously validated with adolescents with ADHD (24), and also not included on the list of adult suggestions. The resulting 83 item survey was sent by email to participants who provided consent to participate in round 2 of the IT.

Following the conclusion of the IT, the winning ideas (see Section 2.5 below) were used to generate a list of skill modules and recommendations for treatment process to enhance the population fit of routine care adult ADHD CBT. We also evaluated whether there were core features of existing adult ADHD CBTs that were noted as neither feasible nor important to participants in the study. These results were used to form a set of best practices for increasing the population fit of CBTs for adult ADHD. This list was subsequently submitted to an additional group of practice experts and adults with ADHD for member checking.

### 2.3 Data integrity

To protect against fraudulent responses, we implemented data integrity measures consistent with best practices for survey security and bot prevention/detection (26–32). We did not provide compensation and directly sent links to limited participant pools. We enabled several Qualtrics features, including ReCAPTCHA fraud detection, RelevantID duplicate detection, Security Scan Monitor, and Prevent Indexing. During manual data review, we screened responses for responses identified by Qualtrics as potential bots (*n* = 0) and verified/removed duplicate responses (*n* = 5).

### 2.4 Measures

#### 2.4.1 Qualitative idea generation survey

The idea generation survey contained three open-ended prompts (1): if you have previously received psychotherapy for your ADHD, what barriers did you experience to feeling satisfied with the therapy? (2), What are some skills, tools, or topics you think are important for providers and clients to discuss during therapy targeting how to manage ADHD symptoms and their impact on daily life?, and (3) What are some things therapists might do during their work with clients to help the process be as ADHD-friendly as possible?

#### 2.4.2 IT survey

The IT survey included the full set of 83 items (42 unique participant suggestions + 41 investigator contributed items). Importance was rated on a scale from -2=relatively unimportant to 2=very important. Feasibility of delivery (practice experts only) was rated from -2=not at all feasible to 2=extremely feasible. Structure of this survey was based on methodology from a previous IT conducted with adolescents with ADHD and their mental health providers (33).

#### 2.4.3 Member checking survey

The member checking survey included the winning practice recommendations as well as the de-implementation suggestions. Respondents were asked to review each practice and answer whether they agreed or disagreed with it. For each practice that was rated as important by adults with ADHD, but not feasible to practitioners, we asked practitioners in the member checking group to share perceived barriers to their implementation.

### 2.5 Analytic plan

#### 2.5.1 Round 1

As a first step, barriers and ideas elicited from the IT’s first prompt were coded qualitatively by two research team members. Responses were coded using a constructivist coding procedure outlined by Merriam (34). Research staff segmented responses into distinct units of data that represented the smallest possible pieces of information that were relevant to the question (i.e., when a respondent generated multiple ideas). The two coders reviewed unique subsets of the data to create categories that were relevant, exhaustive (place all data into a category), and mutually exclusive. The coders gave each category a name that matched its content. Following independent category construction, coders compared the list of categories. The independent coders collaborated to create a final list of categories, each with an operational definition and key examples. In a final step, coders were tasked with reviewing the full set of data and sorting each response using the finalized list of categories and their definitions. All codes were double coded to assess inter-rater agreement, which was 100%.

#### 2.5.2 Round 2

Mean importance and feasibility ratings for each IT idea were calculated and graphed on a scatterplot that was divided into four regions (i.e., Go-zones) (35) that showcased the relative strength of each idea as rated by practice experts. The Go-zone plot’s x-axis and y-axis were crossed at the mean value for each dimension, creating four quadrants: Zone I= high feasibility, high importance; Zone II=high importance, low feasibility; Zone III=low feasibility, low importance, Zone IV=high feasibility, low importance. Ideas that were endorsed as important to adults with ADHD (mean value or higher on the importance scale) are indicated in the Go-zone plot in red typeface. Winning ideas were defined as Zone I ideas that were also rated as having high importance to adults with ADHD.

#### 2.5.3 Round 3

For member checking, percentage endorsement for each recommended practice was examined. Ideas with member checking endorsement at or above 75% were considered acceptable. We also coded the perceived implementation barriers using the same process described above for the idea generation round of the survey.

## 3 Results

### 3.1 Innovation tournament

#### 3.1.1 Perceived barriers

During the IT, adults with ADHD who had received psychotherapy for their ADHD previously (n=9 of 16) generated 6 engagement barriers for adult ADHD psychotherapy: (a) clinician’s low understanding of ADHD—including giving advice that is not well-suited to someone with ADHD (44.4%), (b) lack of attention to relevant impairments (33.3%), (c) experiencing stigma about ADHD from therapist (11.1%), (d) poor coordination between psychotherapist and medication provider (11.1%), (e) forgetfulness with therapy homework (11.1%), (f) difficulty scheduling appointments (11.1%), and (g) therapist’s fixation on their own ADHD (11.1%).

#### 3.1.2 Idea generation

**Table 2** displays each of the 42 unique ideas emerging from the IT as well as the 41 ideas contributed by the common elements of adult ADHD CBT literature review and tailored MI for ADHD strategies. **Table 2** also notes the source of each idea as well as means and standard deviations for importance ratings and feasibility ratings provided by the adult ADHD validation sample (*N=*53) and practice experts (*N=*16). Go-zone plot (see **Figure 1**) indicated high perceived importance among adults with ADHD and practice experts, as well as feasibility among adults with ADHD for 14 content areas and 18 aspects of therapeutic process (38.6% of the generated ideas).

**Table 2:** Importance and feasibility ratings for IT ideas.

Idea	Idea Source	Adult ADHD Importance	Practice Expert Importance	Practice Expert Feasibility
1. Organizational systems	CBT-C	1.19 (0.96)	1.56 (0.51)	1.31 (1.01)
2. Planning skills	CBT-C	1.21 (0.99)	1.88 (0.34)	1.88 (0.34)
3. How to get support from a family member	CBT-C	0.92 (1.03)	1.06 (0.85)	0.88 (1.09)
4. Managing multiple tasks	CBT-C	1.15 (0.91)	1.56 (0.63)	1.38 (0.72)
5. Keeping track of important items and managing my stuff	CBT-C	1.17 (1.10)	1.56 (0.63)	1.38 (0.72)
6. Managing overwhelming tasks	CBT-C	1.66 (0.61)	1.94 (0.25)	1.44 (0.73)
7. Teaching how to be less distracted	CBT-C	0.75 (1.05)	1.13 (1.09)	0.38 (1.45)
8. Learn how to manage hyperfocus	CBT-C	1.04 (1.00)	1.25 (0.68)	0.94 (0.77)
9. Modifying or designing one’s optimal environment	CBT-C	1.17 (0.96)	1.56 (0.51)	1.00 (1.15)
10. Learning to think about ADHD more adaptively	CBT-C	1.38 (0.63)	1.81 (0.40)	1.69 (1.01)
11. Anti-procrastination strategies	CBT-C	1.25 (0.90)	1.88 (0.34)	1.44 (1.03)
12. Recognizing when you slip-up and how to re-engage	CBT-C	1.25 (0.83)	1.69 (0.48)	1.44 (0.81)
13. Problem-solving skills	CBT-C	0.83 (1.05)	1.56 (0.51)	1.44 (0.73)
14. Rewarding oneself to help with motivation	CBT-C	0.57 (0.97)	1.19 (1.11)	1.19 (1.10)
15. Building a toolbox of self-motivation strategies	CBT-C	1.23 (0.72)	1.50 (0.82)	1.50 (0.89)
16. Teaching mindfulness/calming the mind	CBT-C	0.96 (0.98)	1.00 (1.21)	0.94 (1.00)
17. Self-monitoring one’s behavior	CBT-C	1.02 (0.95)	1.69 (0.60)	1.06 (0.85)
18. Healthy routines and lifestyle behaviors	CBT-C	0.94 (1.03)	1.63 (0.81)	0.69 (1.30)
19. Interpersonal or communication skills	CBT-C	0.94 (1.26)	1.25 (1.00)	0.63 (1.26)
20. Self-advocacy skills or leveraging resources	CBT-C	1.19 (0.76)	1.56 (0.51)	1 (1.03)
21. Working on self-worth/self-concept	AA	1.53 (0.82)	1.38 (0.89)	1.25 (0.93)
22. Learning how to respond to negative thoughts and emotions	AA	1.57 (0.64)	1.93 (0.25)	1.63 (0.81)
23. Learning how to respond to thoughts and emotions that get the person off track	AA	1.51 (0.64)	1.69 (0.60)	1.25 (1.06)
24. Reducing impulsivity	AA	0.92 (1.14)	1.19 (0.83)	0.19 (1.05)
25. Use of goal setting as a motivation strategy	AA	0.70 (0.87)	1.44 (0.73)	1.13 (0.89)
26. Learning to relax be still/reduce restlessness	AA	0.57 (1.18)	0.38 (1.41)	0.19 (0.83)
27. Reducing shame and nurturing confidence	AA	1.53 (0.77)	1.44 (0.73)	0.88 (1.15)
28. Increasing self-acceptance	AA	1.55 (0.75)	1.56 (0.51)	1.31 (0.87)
29. Learning to actively listen to others	AA	0.81 (1.06)	1.19 (0.91)	0.69 (1.08)
30. Learning how to connect with others in the ADHD community	AA	0.57 (1.21)	1.00 (1.21)	0.75 (1.06)
31. Managing rejection sensitivity	AA	1.42 (0.80)	1.13 (1.02)	0.75 (0.93)
32. Self-exploring strengths and weaknesses	AA	1.17 (0.83)	1.50 (0.89)	1.44 (0.96)
33. Self-awareness of masking ADHD symptoms	AA	1.25 (0.87)	0.5 (1.32)	0.44 (1.15)
34. Building an inventory of coping skills for ADHD	AA	1.45 (0.64)	1.44 (0.81)	0.94 (1.12)
35. How to find jobs or situations that fit strengths	AA	1.26 (0.88)	1.06 (1.06)	0.44 (1.36)
36. Addressing time blindness/how to be on time for things	AA	0.91 (1.02)	1.69 (0.60)	1.13 (1.20)
37. How to prioritize tasks	AA	1.19 (0.94)	1.88 (0.34)	1.56 (0.89)
38. Using a planner	AA	0.15 (1.31)	1.75 (0.45)	1.00 (1.10)
39. Task initiation skills	AA	1.25 (0.78)	1.81 (0.40)	1.44 (0.89)
40. Self-administering effective reminders	AA	0.70 (1.03)	1.63 (0.50)	1.13 (1.02)
41. Emotion regulation skills	AA	1.58 (0.82)	1.81 (0.40)	1.25 (1.00)
42. Identifying and practicing hobbies/pleasurable activities	AA	0.74 (1.13)	1.13 (0.96)	1.19 (0.91)
43. Psychoeducation on science of ADHD	MI	0.89 (1.17)	1.56 (0.63)	1.44 (0.89)
44. Understanding ADHD medications	MI	0.92 (1.14)	1.5 (0.82)	1.19 (1.11)
45. Therapist offers compassion and teaches self-compassion	MI	1.47 (0.72)	1.56 (0.73)	1.44 (0.96)
46. Client gets to make individual choices on therapy topics	MI	0.96 (1.00)	1.00 (1.10)	0.69 (1.49)
47. Client and therapist set agenda collaboratively	MI	1.26 (0.94)	1.81 (0.40)	1.63 (0.89)
48. Therapist provides direct instruction and advice on how to do things	CBT-P	0.38 (1.08)	0.63 (0.81)	0.94 (0.93)
49. Therapist allows client to self-explore best way for them do things	MI	0.85 (0.97)	1.31 (0.87)	1.06 (0.93)
50. Client and therapist work together to identify creative solutions	MI	1.58 (0.66)	1.50 (0.89)	1.44 (0.89)
51. Spending extra time planning out attempts to try new skills and routines	MI	0.66 (1.02)	1.38 (0.89)	0.75 (1.34)
52. Extra time in session time to review attempts to try out new skills and routines	MI	0.83 (0.96)	1.38 (1.09)	0.75 (1.39)
53. Therapist is strengths based and affirmative	MI	1.21 (0.82)	1.56 (0.63)	1.38 (1.09)
54. Therapist is empathic to ADHD-related struggles with follow through	MI	1.64 (0.65)	1.63 (0.62)	1.31 (1.08)
55. Therapist will confront and call out the client for not following through when needed	CBT-P	0.53 (1.31)	0.75 (1.39)	1.00 (1.15)
56. Provides basic education on ADHD	CBT-P	0.81 (1.06)	1.94 (0.25)	1.88 (0.5)
57. Helps client explore their goals and values	MI	1.45 (0.64)	1.69 (0.60)	1.56 (0.89)
58. Helps client explore how ADHD relates to their identity	MI	1.34 (0.85)	1.13 (0.89)	1.00 (1.10)
59. Work on long-term goal setting	MI	1.15 (0.79)	1.75 (0.44)	1.19 (0.98)
60. Treatment pace can be adjusted to the needs of the client	MI	1.45 (0.72)	1.69 (0.60)	0.69 (1.25)
61. Allowing treatment to be tailored to changes that would be most meaningful to client	MI	1.55 (0.64)	1.88 (0.34)	1.25 (0.93)
62. Helping with habit formation	MI	1.09 (0.80)	1.69 (0.48)	1.19 (0.91)
63. Therapist gives client a high level of choice in topics and action plans	MI	0.77 (1.03)	0.44 (1.31)	0.44 (1.31)
64. Therapists helps client walk through exactly how they will complete skill practice plans—including problem-solving barriers in advance.	MI	0.77 (1.15)	1.38 (0.96)	0.88 (1.41)
65. Pre-set session topics each week based on a standardized treatment that has shown research evidence	CBT-P	-0.19 (1.21)	0.63 (1.09)	0.56 (1.36)
66. Completing ADHD symptom measures each week with therapist to discuss improvement in symptom severity	CBT-P	0.04 (1.36)	-0.19 (1.22)	0.44 (1.31)
67. Checking in on personal goals each week to notice positive steps	MI	0.75 (0.92)	1.69 (0.48)	1.63 (0.81)
68. Therapist is judgement free when client struggles to uphold commitments	MI	1.47 (0.93)	1.31 (1.01)	1.25 (0.93)
69. Therapist avoids shaming and blaming	MI	1.75 (0.65)	1.88 (0.50)	1.56 (0.81)
70. Therapist promotes a growth rather than fixed mindset about ADHD	AA	1.75 (0.55)	1.69 (0.87)	1.50 (1.03)
71. Therapist carefully and explicitly explains things to client	AA	1.23 (0.90)	1.5 (0.73)	1.38 (0.81)
72. Therapist asks open questions to client to help them open up	AA	1.25 (0.98)	1.69 (0.48)	1.56 (0.81)
73. Hands on activities are done in session	AA	0.60 (1.01)	1.63 (0.62)	1.13 (0.96)
74. Extra attention is given to making therapy tasks fun	AA	0.23 (1.07)	0.5 (1.26)	0.13 (1.15)
75. Therapist offers incentives to the client for engagement	AA	-0.26 (1.09)	-0.19 (1.28)	-0.44 (1.09)
76. Plans are kept as simple as possible	AA	0.68 (1.01)	1.31 (0.79)	1.06 (1.12)
77. Therapist provides resources that can be referenced outside session	AA	1.36 (0.62)	1.31 (1.25)	1.44 (0.96)
78. Therapist offers reminders to client throughout the week	AA	0.17 (1.19)	0.38 (1.45)	-0.75 (1.24)
79. Therapist offers appointment reminders	AA	1.11 (1.03)	1.19 (1.22)	0.81 (1.22)
80. Flexibility offered on no-shows/being late to appointments	AA	0.32 (1.24)	-0.19 (1.47)	-0.56 (1.21)
81. Therapist should have ADHD themselves	AA	-0.11 (1.40)	-1.31 (1.14)	-1.31 (1.01)
82. Therapist should have a deep understanding of ADHD	AA	1.87 (0.34)	1.75 (0.45)	1.13 (1.15)
83. Encourages client to use trial and error, even if therapist doubts an idea will work	AA	0.72 (1.08)	1.13 (1.02)	0.94 (1.00)

AA= an idea suggested by an adult with ADHD, CBT-C= common content element of cognitive behavioral therapy for adult ADHD included by the research team, CBT-P=common process element of cognitive behavioral therapy for adult ADHD included by the research team, MI=ADHD-specific adaptation or emphasis of Motivational Interviewing included by the research team.

**Figure 1:**
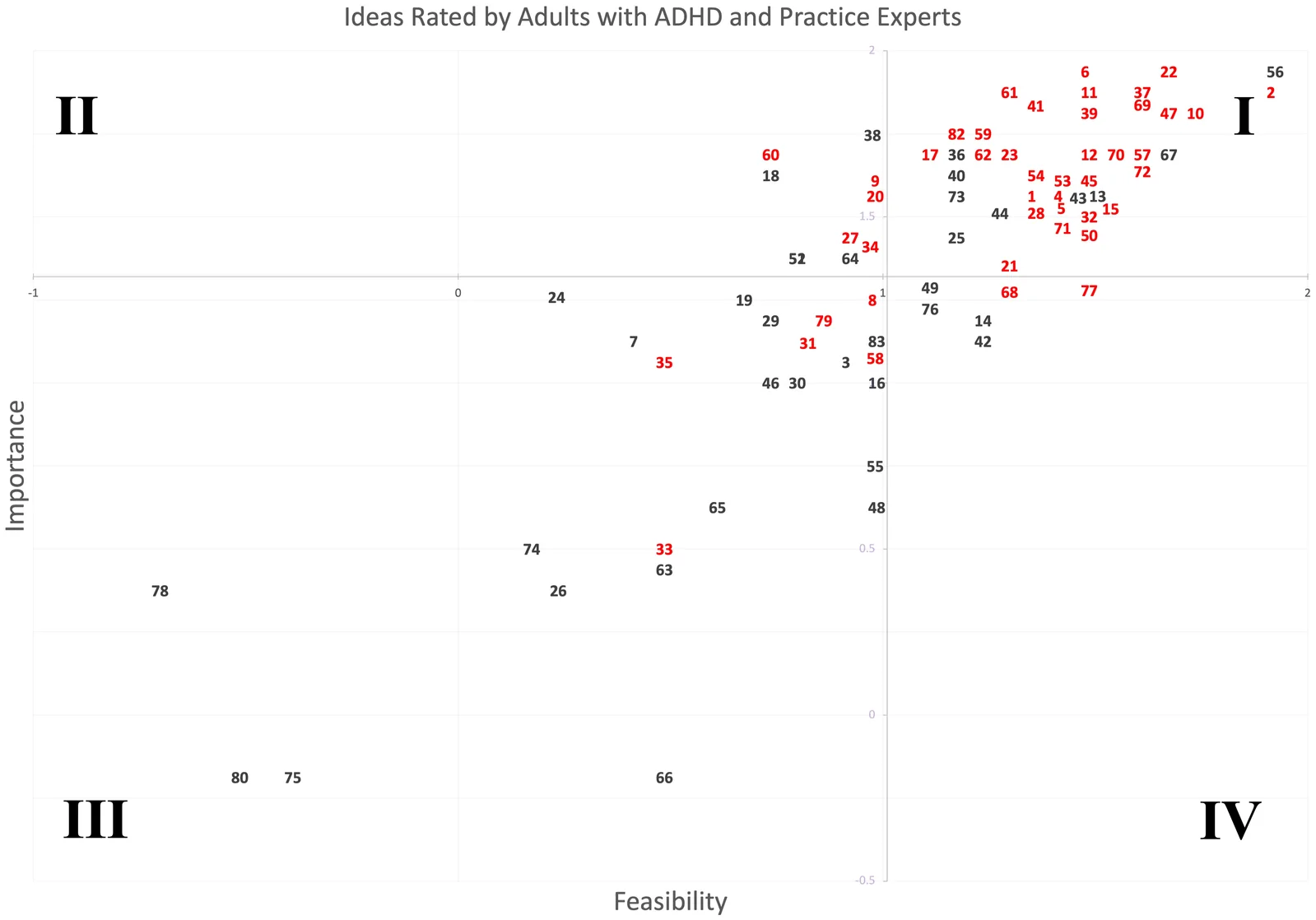
Go zone plot for participant-rated implementation strategies. The x-axis and y-axis cross at the mean value for each dimension. Zone I= high feasibility, high importance; Zone II=high importance, low feasibility; Zone III=low feasibility, low importance, Zone IV=high feasibility, low importance. Red values represent ideas voted as high importance by adults with ADHD.

#### 3.1.3 Feasibility and importance rankings

Ranked in terms of highest cumulative feasibility and importance (see **Table 2**), results indicated the top skill focal areas perceived to be important to both adults with ADHD and practice experts (and feasible to experts) were developing strategies for: (a) task management (i.e., #6 managing overwhelming tasks, #39 task initiation, #4 managing multiple tasks), (b) organization (i.e., #1 organization systems, #5 keeping track of important items and managing my stuff), #2 planning, and #37 prioritization, (c) emotion regulation (i.e., #41 emotion regulation skills, #22 learning to respond to negative emotions, #23 learning how to respond to emotions that get me off track), (d) responding to negative/maladaptive thoughts (i.e., #10 learning to think about ADHD more adaptively, #22 learning to respond to negative thoughts, #23 learning to respond to thoughts that get me off track), (e) #11 anti-procrastination/#15 self-motivation strategies, and (f) #59 long-term goal setting.

#### 3.1.4 Winning strategies

Regarding therapy process 18 strategies were endorsed by both adults with ADHD and practice experts, with 17 representing elements of MI (see **Table 2**) and one idea related to therapist competence: #82 that the therapist should have high knowledge of ADHD. ADHD-adapted MI-consistent elements were related to how therapists support debriefing on follow-through challenges and successes (i.e., #12 recognizing when you slip-up and how to re-engage, #62 discussing habit formation, #17 self-monitoring one’s behavior), strength-based framing (i.e., #53 therapist is strengths based and affirmative; #69 therapist avoids shaming and blaming, #21 working on self-worth/self-concept, #28 increasing self-acceptance, #70 therapist promotes a growth rather than fixed mindset about ADHD), guiding a process of self-exploration (i.e., #32 self-exploring strengths and weaknesses, #57 helps client explore their goals and values), expressing MI Spirit (i.e., #45 therapist offers compassion and teaches self-compassion, #50 client and therapist work together to identify creative solutions, #54 therapist is empathic to ADHD-related struggles with follow-through), #61 allowing treatments to be tailored to changes that would be most meaningful to the client, #71 giving didactic information simplistically (i.e., therapist carefully and explicitly explains things to clients), and #72 therapists asks open ended questions to client to help them open-up.

#### 3.1.5 Candidates for de-implementation

In Zone III, there also were some existing elements of manualized CBTs for adult ADHD that may be candidates for de-implementation based on being rated both of low importance and low feasibility by participants. In particular, #66 completing ADHD symptom screeners each week was rated over one standard deviation below the mean for importance (adults with ADHD and practice experts) and feasibility. Other Zone III skills content practices that were also rated low by adults with ADHD included #3 how to get support from one’s family, #7 teaching the adult how to be less distracted, #16 teaching mindfulness/calming the mind, and #19 working on interpersonal/communication skills. Other existing CBT process practices that were rejected by participants included #48 providing direct instruction and advice to the adult, #55 confronting clients when they do not complete homework assignments, and #65 having pre-set session topics each week based on a standardized treatment manual. One MI practice--#46 giving the client individual choice about session topics and plans-–also fell into this category (collaborative agenda setting was preferred).

#### 3.1.6 Ideas with low feasibility

There were also several practices that adults with ADHD rated as important, but practice experts rated as not feasible for them to implement (Zone II or Zone III). In particular, adults with ADHD rated #27 reducing shame and nurturing confidence (Zone II) as over one standard deviation above the mean for importance, but this focal area was not viewed as feasible to practice experts (although practice experts also rated it as important). Ten other low feasibility practices were rated as important to adults with ADHD. In Zone II (high importance, low feasibility to practice experts): (a) #9 modifying or designing one’s optimal environment, (b) #20 self-advocacy skills or leveraging resources, (c) #34 building an inventory of coping skills for ADHD, (d) #60 adjusting treatment pace to the needs of the client. In Zone III (low importance, low feasibility to practice experts): (e) #8 learning to manage hyperfocus, (f) #31 managing rejection sensitivity, (g) #33 self-awareness of masking ADHD symptoms, (h) #35 finding jobs or situations that fit one’s strengths, (i) #58 exploring how ADHD relates to identity (6), offering appointment reminders to the client.

### 3.2 Member checking

**Table 3** lists the practice recommendations and de-implementation recommendations that emerged from this study and were submitted for member checking, along with the percentage of the member checking sample that agreed with each. Of the 27 ideas submitted for member checking, 23 met the acceptability threshold. Four ideas that did not were all related to de-implementation—*de-implementing:* (a) distractibility reduction training (58.8% endorsed), (b) mindfulness strategies (58.8%), (c) interpersonal skills training (52.9%), and (d) a style that gives clients individual authority in treatment (64.9%; see **Table 3**). **Table 4** lists perceived implementation barriers generated by the member checking sample for each practice that was rated in round 2 as important by adults with ADHD, but not feasible to practitioners.

**Table 3:** Member checking sample’s endorsement of practice recommendations.

Recommendation	% Endorsed
1. A focus on task management skills	94.1
2. A focus on organization, planning, and prioritization	88.2
3. A focus on emotion regulation skills	94.1
4. A focus on responding to negative/maladaptive thoughts	94.1
5. A focus on anti-procrastination and self-motivation strategies	82.4
6. A focus on long-term goal setting	82.4
7. Therapist should have a high understanding of ADHD	94.1
8. The should be a focus on habit formation	76.4
9. Shortcomings and forgetfulness should be reframed as opportunities to gain wisdom about the self	82.4
10. Progress monitoring should occur on individualized goals rather than the DSM-5 symptoms	82.4
11. Therapy should take a strength-based framing	94.1
12. There should be a focus on self-acceptance and building self-efficacy and self-confidence	88.2
13. The therapist should guide the client to self-explore their goals, values, and priorities	94.1
14. The therapist should demonstrate a collaborative and egalitarian style	88.2
15. The therapist should express a high level of empathy for the client with ADHD	94.1
16. A style that supports the autonomy of the client should be adopted	94.1
17. Didactic information and teaching should occur in very small chunks	82.4
18. The therapist should ask open-ended questions to help the client open up about their experiences	94.1
19. Therapists should consider *not* having their clients complete ADHD screening questionnaires each week.	94.1
20. Therapists should *avoid* strict, standardized treatment protocols in favor of individualized or eclectic approaches that draw from evidence based practices but are tailored to clients.	88.2
21. Therapists should *avoid* a style that gives the client individual authority (rather than joint decision making with the therapist) on session topics and plans	64.7
22. Therapists can consider *skipping* content on how to get support from one’s family	76.4
23. Therapists can consider *skipping* content on teaching the adult how to be less distracted	58.8
24. Therapists can consider *skipping* the teaching of mindfulness strategies	58.8
25. Therapists can consider skipping content on interpersonal and communication skills	52.9
26. Therapists should *avoid* providing direct instruction and advice to clients without permission	88.2
27. Therapists should *avoid* confronting or calling out clients when they do not complete their therapy homework.	82.4

**Table 4:** Member checking: perceived barriers to implementation of important practices.

Practice	Perceived barriers
Reduce shame and nurture confidence	Low practitioner knowledge of how ADHD relates to shame; low practitioner skill in this area
Manage hyperfocus	Low practitioner knowledge of ADHD hyperfocus; low practitioner skill in this area; may see this as a target for medication
Modify or design optimal environment	Low practitioner knowledge of environments that help people with ADHD; low practitioner skill in this area; too much effort for practitioner; relies on client implementation
Increase self-advocacy/leverage resources	Low practitioner knowledge of available resources; low practitioner skill in this area; not interesting to practitioner; too much effort for practitioner
Manage rejection sensitivity	Low practitioner knowledge of ADHD and rejection sensitivity; low practitioner skill in this area; practitioner rejection of this concept; desire to protect client from feeling rejected
Increase self-awareness of masking	Low practitioner knowledge of ADHD and masking; low practitioner skill in this area; client discomfort with this topic; practitioner rejection of this concept
Build and inventory of ADHD coping skills	Low practitioner skill in this area; low client follow-through
Find jobs/situations that fit strengths	Low practitioner knowledge of ADHD and environmental fit; Outside the scope of practice; low practitioner skill in this area;
Explore how ADHD relates to identity	Low practitioner knowledge of ADHD and identity issues; low practitioner skill in this area; insufficient time; theoretical orientation of practitioner views ADHD as external to self
Adjust treatment pace to client needs	Low practitioner skill in this area; insurance constraints; systems of care constraints; lack of awareness of client needs; too much effort for practitioner
Offer appointment reminders	Outside the scope of practice; technology challenges; desire for patient to maintain individual responsibility; too much effort for practitioner

## 4 Discussion

After completing the IT, we identified: (a) six content areas for CBT that felt particularly relevant to adults with ADHD and were found to be equally valued and feasible to practice experts, (b) 18 process practices that might improve therapeutic engagement for adults with ADHD that were endorsed by both adults with ADHD and practices experts, (c) nine existing practices that could be considered for de-implementation based on being rejected by both adults with ADHD and practice experts, (d) five practices that may require the development of better practitioner implementation supports, given that they were important to adults with ADHD and practitioners, but rated by practitioners as non-feasible to implement, and (e) six practices that require additional exploration as they were rated as important to adults with ADHD but neither important nor feasible to practice experts. We elaborate on these findings below.

The 14 recommended practices spanning six content areas that were identified as highly valued by adults with ADHD and practitioners generally represented EF skills training, but also, targets of cognitive restructuring (e.g., managing negative thoughts and emotions, anti-procrastination strategies). These elements are consistent with the components of existing CBT manuals for adult ADHD, including some linked to greater improvements in ADHD symptoms in meta-analyses (11, 12) and were also endorsed highly by the member checking sample. The finding that adults with ADHD valued the existing content of CBTs for adult ADHD (11) is contextually consistent with the finding that only 1/3rd of therapy recipients in the idea generation sample found that the psychotherapy they received was ill-tailored to their challenges. Instead, barriers largely included process-oriented complaints about the way therapy was delivered.

Chief among these complaints was a need to ensure that therapists have high knowledge of adult ADHD, which was also the process element most valued by participants. Previous research has indicated that routine care CBT providers may not have specialized training or up to date knowledge in adult ADHD (15, 16). Our member checking sample also frequently cited low practitioner knowledge as a practice implementation barrier (see **Table 4**). Thus, systemic efforts are needed to increase adult ADHD education and training in psychotherapy workforces. The ADHD-specific adaptations of MI that were presented to participants also faired very well in the IT, with 17 receiving high importance and feasibility ratings. The member checking sample also strongly endorsed corresponding practice recommendations (see **Table 3**). Based on these findings, we suggest development and testing of an MI-enhanced CBT for adults with ADHD, perhaps modeled after existing adolescent manuals (19–22). The specific recommendations in this investigation (see **Tables 2**, **3**) might guide the nature of such a treatment.

Besides, failure to implement engagement-focused approaches, therapist use of techniques that are a poor population fit also may lead to engagement challenges. There were nine practices that both practitioners and adults with ADHD rated as having below average importance and feasibility—making them candidates for de-implementation. Five out of nine survived member-checking (see **Table 3**) including several practices that are routine in manualized CBTs for adults with ADHD such as getting weekly ratings of ADHD symptom severity, discussing how to solicit supports from family members, adopting a didactic style, and using standardized treatment manuals (6–11). Removal of skills training in distractibility reduction, mindfulness, and communication, as well as giving clients individual authority, demonstrated mixed endorsement by the member checking sample (52.9%-64.7%). Additional work is needed to understand mixed opinions on these practices. Future iterations of CBT for adults with ADHD might consider omitting these practices in favor of replacements that are a better population fit, such as using idiographic measures of progress, modular, individualized session structures, offering a collaborative style, and sticking to topics that are valued highly by patients (**Table 2**).

We note that there also were practices that clinicians felt were not feasible to implement, despite feeling they were important. These included nurturing shame and self-confidence, helping adults with ADHD optimize their environment, and teaching self-advocacy skills. Therapists also rated below average feasibility for creating an inventory of coping skills for ADHD and adjusting treatment pace to the needs of the client—despite these being areas that were important to participants. In our member checking survey, we queried perceived barriers to implementing these practices and learned that low practitioner knowledge and skills were the primary potential roadblocks cited by the sample. Member checking participants also shared a belief that practitioner opposition to topics (such as building self-awareness of masking, managing rejection sensitivity) also may influence their implementation along with systemic and scope of practice barriers (e.g., not being able to slow the pace of treatment due to healthcare system requirements or support job searching as a billable treatment).

This study has several limitations. We prioritized the external validity of our sample (i.e., generalizability to real world consumers of adult ADHD treatment and their routine care practitioners) at a cost to internal validity (i.e., confirming that adults with ADHD met current DSM-5 criteria for ADHD at the time of their participation). Much like the help-seeking population for adult ADHD in the U.S (36)., our sample was primarily White, middle-aged, and female. While this makes our findings generalizable to the average consumer of adult ADHD treatments, they may not generalize to populations who are male, non-White, and younger or older ages. In the future, it will be important to specifically member check these results with individuals outside the average demographic of this sample. As part of this work, considerations of gender-specific treatment modules and modules that address comorbidities beyond ADHD’s core symptoms and impairments is critical (37). In considering the limitations of this study, it is also important to acknowledge the limitations of CBT—which unlike medications for ADHD—do not directly impact cognition and instead teaching cognitive and behavioral skills to compensate for underlying neuropsychological difficulties. Thus, combined medication and CBT is currently recommended for adults with ADHD.

All in all, it appears that there are several steps forward stemming from this work. First, there should be efforts to develop practice materials and training/educational tools that can facilitate practitioner application of the co-designed best practices in adult ADHD CBT identified herein. Co-design efforts should continue through the resource development phase. Research is needed on both the implementability and effectiveness of a CBT package that is based on the findings of this study. We also wish to ascertain the extent to which best practices are applied in routine care CBT for adults with ADHD to identify specific areas for implementation and de-implementation initiatives. Ultimately, work is needed to understand if engagement in CBT for adults with ADHD in routine care settings can be enhanced by integrating the practices identified in the present investigation. In the meantime, clinicians are welcome to use the 83 practices collected in this study to assess individual client preferences for care.

## Data Availability

The raw data supporting the conclusions of this article will be made available by the authors, without undue reservation.
